# Supplementary material for: Multinuclear non-haem iron-dependent oxidative enzymes: landscape of their substrates, partner proteins and biosynthetic gene clusters
Source: Microb Genom. 2025 Jul 31;11(7):001462. doi: 10.1099/mgen.0.001462 (PMC12452194; doi:10.1099/mgen.0.001462)
Supplement: Uncited Supplementary Material 1. [file mgen-11-01462-s001.pdf]

Supplementary Material for:

Multinuclear non-heme iron dependent oxidative enzymes: Landscape of their substrates, partner proteins and biosynthetic gene clusters

R. Antoine,[a#] L. Leprevost,[a] S. Jünger,[b] S. Zirah,[b] G. Lippens,[c]
Y. Li,[b] S. Dubiley,[c] F. Jacob-Dubuisson[a#]

Table S1. Protein domains genetically associated with DUF692 genes

Pfam_TIGRfam_Name	Count	% co-occurrence	
DUF692	13976	102	
DUF2063	9696	71	
DoxX	5414	40	
DUF2282	5268	39	
TIGR02937	3116	23	RNA polymerase sigma-70 family
Sigma70_r2	3078	23	
Sigma70_r4_2	2878	21	
ABC_tran	2708	20	
Response_reg	2394	18	
HATPase_c	1694	12	
NrsF	1596	12	
HTH_1	1471	11	
LysR_substrate	1466	11	
Pyr_redox_2	1442	11	
TIGR04222	1438	11	
MFS_1	1338	10	
BPD_transp_1	1046	8	
HisKA	1024	8	
TetR_N	917	7	
Pyr_redox_dim	908	7	
HTH_18	850	6	
GGDEF	846	6	
TIGR00254	810	6	
EamA	795	6	
HAMP	760	6	
CBS	748	5	

The absolute numbers and percentages of genetic association between these protein domains and MNIO enzymes are provided. The list includes only the proteins or domains coded in the MNIO loci at frequencies of at least 5%. A proprotion greater than 100% is explained by the fact that tandem MNIO genes are present in some BGCs. In red are the domain signatures already known to be found in MNIO-encoding BGCs. The relevant urls are (https://www.ebi.ac.uk/interpro/entry/pfam/PF07681) for DoxX, (https://www.ebi.ac.uk/interpro/entry/pfam/PF08281) for Sigma-70 region 4, (https://www.ebi.ac.uk/interpro/entry/pfam/PF04542) for Sigma-70 region 2 and (https://www.ebi.ac.uk/interpro/entry/pfam/PF06532) for NrsF. Note that not all genetically associated proteins are necessarily involved in RiPP biosynthesis. In particular, the signatures 'ABC_tran', 'Response_reg' and 'HATPAse_c' were not considered relevant, because most bacterial genomes harbor numerous genes coding for paralogs of ABC transporters and two-component systems. The MbnC signature was not found in significant proportions because the methanobactins BGCs represent a very small number of all BGCs collected.

Table S2. Potential precursors genetically associated with MNIO enzymes of the largest sequence cluster shown in Fig. 1B.

Number of Cys	counts
1 Cys	27
2 Cys	93
3 Cys	217
4 Cys	107
5 Cys	92
6 Cys	92
7 Cys	39
8 Cys	37
9 Cys	32
10 Cys	17
11 Cys	14
12 Cys	4
13 Cys	3
14 Cys	0
15 Cys	2
Total	776

Only putative precursors encoded next to MNIO genes and that do not belong to the previously described families were included.

Table S3. Accession numbers of all proteins identified in this work (Excel file)

Table S4. Analysis of complete genomes: genome identification, accession numbers of the proteins of interest, and numbers of paralogs in genome (Excel file)

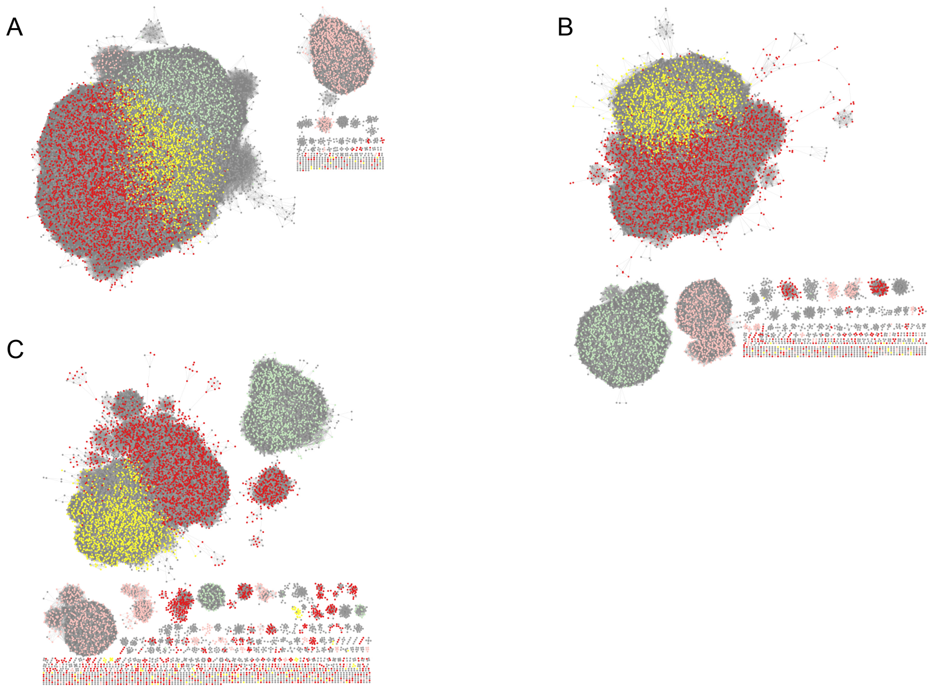

Figure S1. SSN analyses. Representative node network of MNIO enzymes with alignment score thresholds of 75, (A) 85 (B) and 95 (C; pairwise identities of 50%, 55% and 60%, respectively). MNIO enzymes genetically associated with Buf1, Buf2, Buf_EGKCG/oxazolins and TIGR04222 proteins are colored red, yellow, light green and pink, respectively.

Fig3G

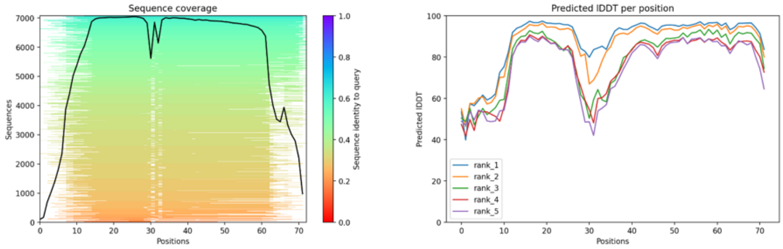

Fig3H_1

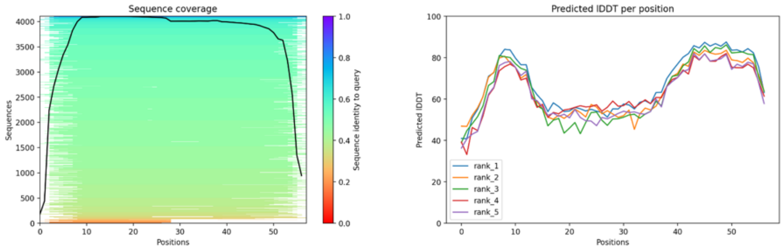

Fig3H_2

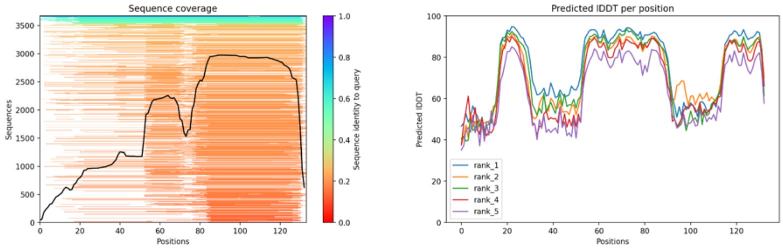

Fig3I

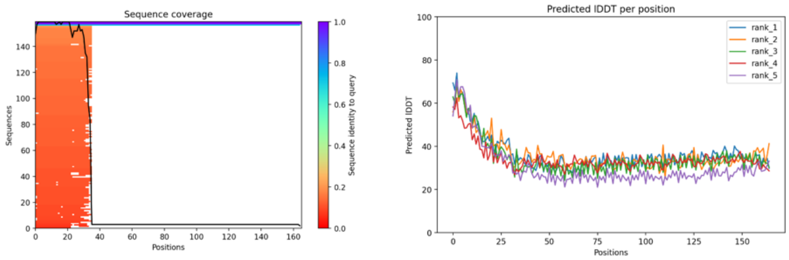

Fig4D_1

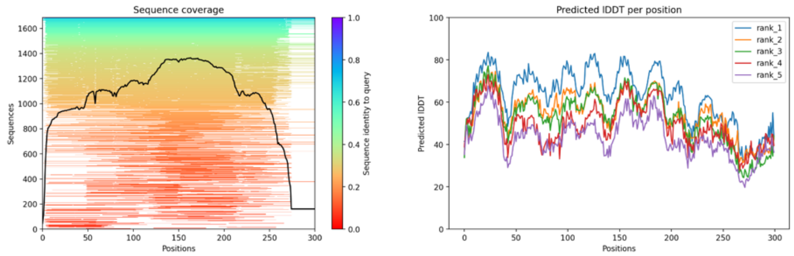

Fig4D_2

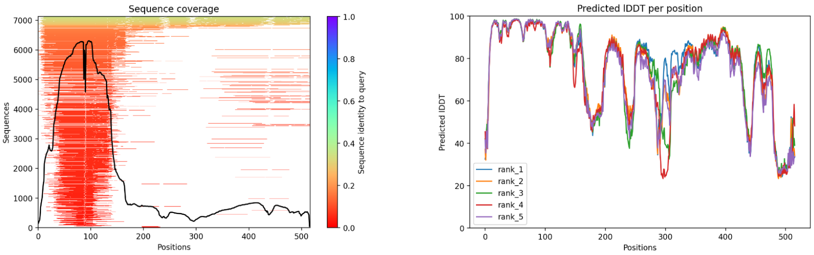

Fig5A_1

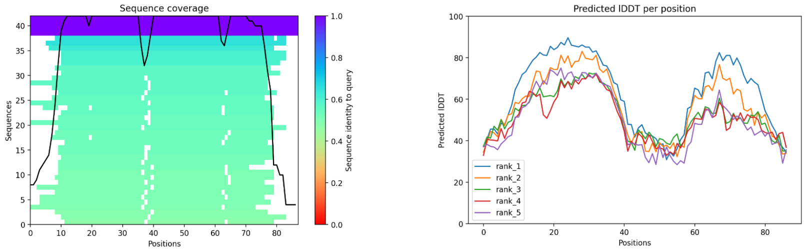

Fig5B_1

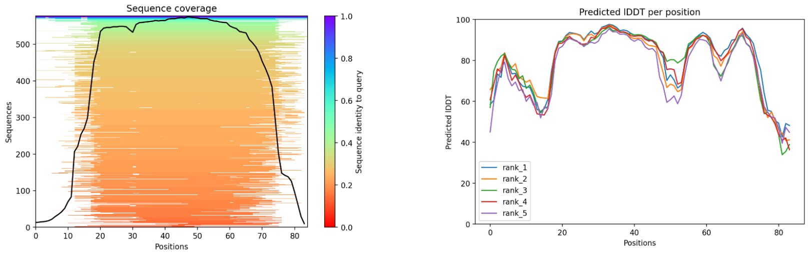

Fig6a

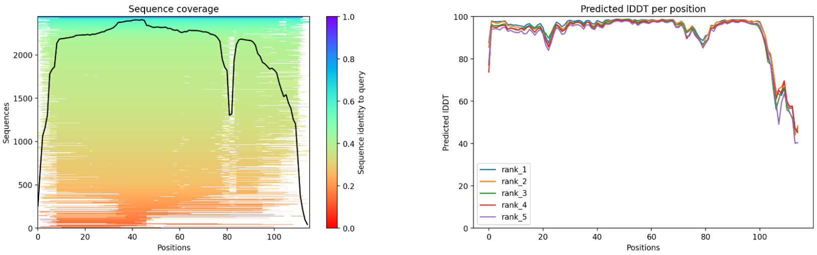

Fig6b

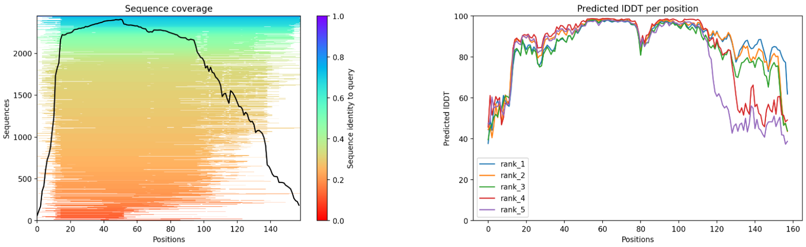

Fig6c

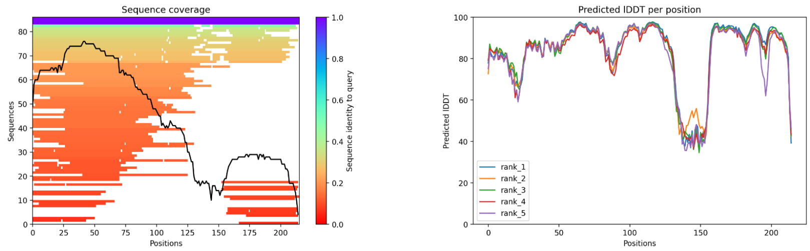

Fig6d

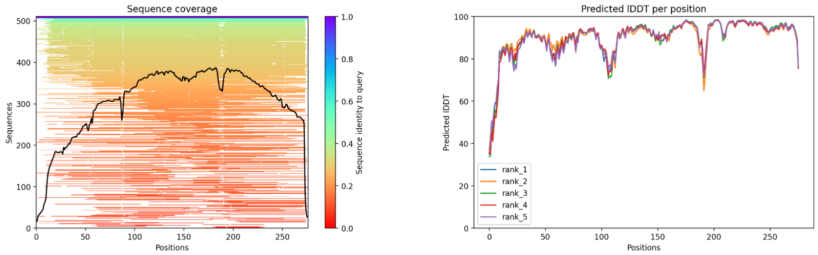

Figure S2. Analyses of the structural models. The sequence coverage and the predicted local distance difference test scores for all positions in the sequences are provided for the Alphafold models shown in the main figures, with the numbers corresponding to those of the figures.

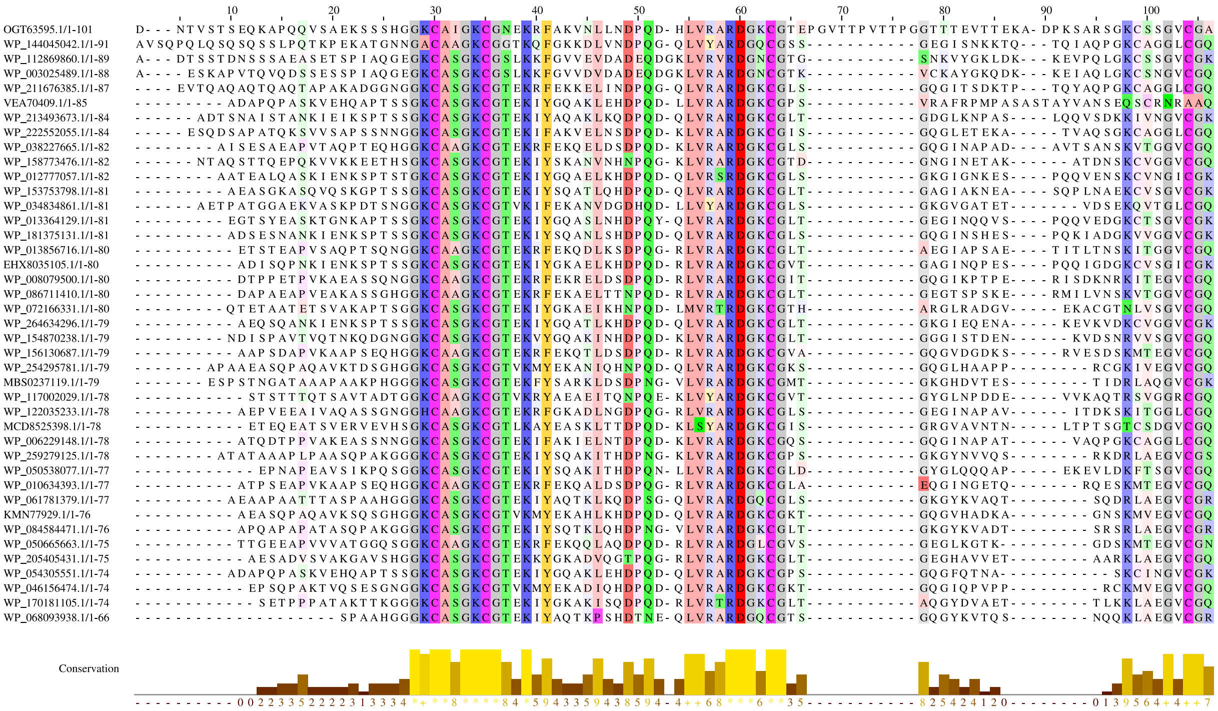
Conservation

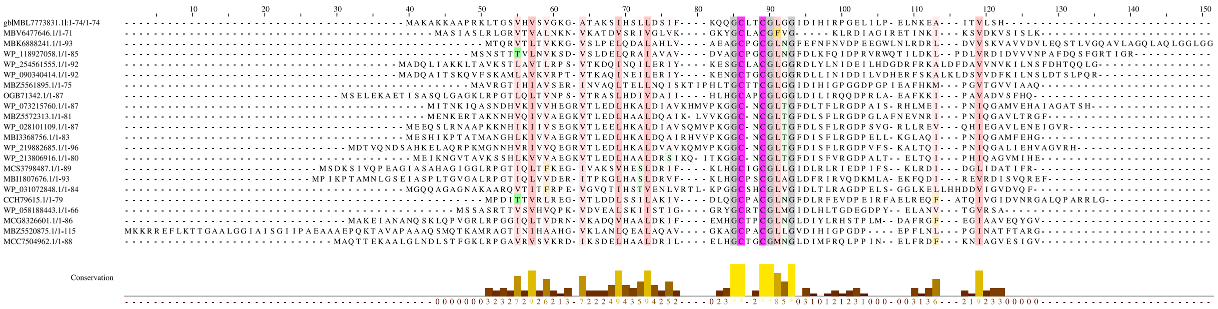
Conservation

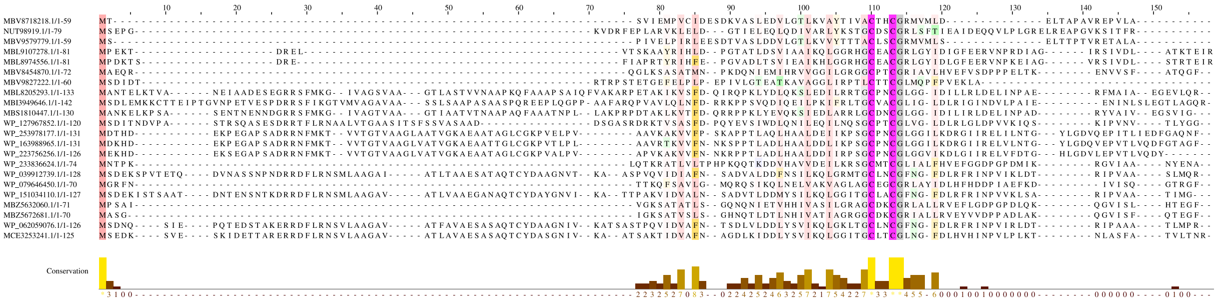
Conservation

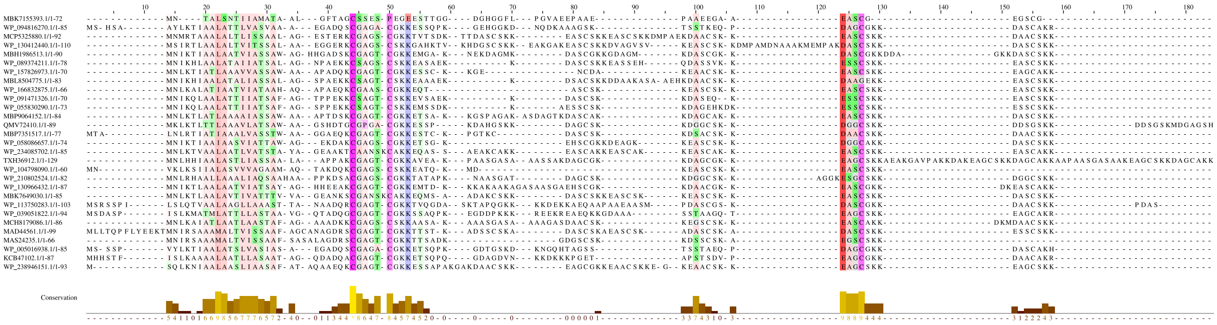

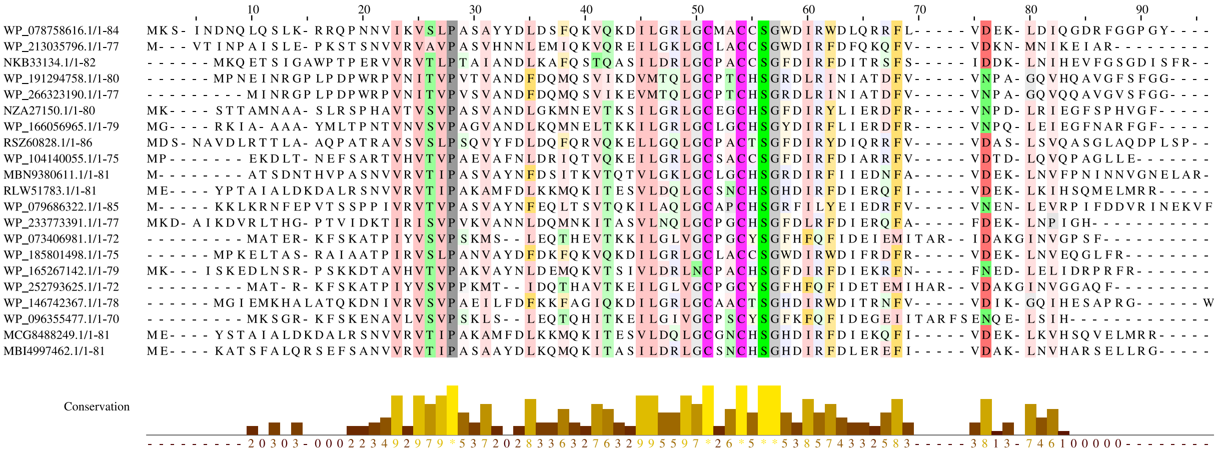
10 20 30 40 50 60 70 80 90
WP_078758616.1/1-84 MKS-INDNQLQSLK-RRQPNNVIKVSLPASAYYDLDSFQKVQKDILGRLGCMACCSGWDIRWDLQRRFL-----VDEK-LDIQGDRFGGPGY----
WP_213035796.1/1-77 M---VTINPAISLE-PKSTSNVVRVAVPASVHNNLEMIQKVQREILGKLGCLACCSGWDIRFDFQKQFV-----VDKN-MNIKEIAR---------
NKB33134.1/1-82 ------MKQETSIGAWPTPERVVRVTLPTAIANDLKAFQSTQASILDRLGCPACCSGFDIRFDITRSFS-----IDDK-LNIHEVFGSGDISFR--
WP_191294758.1/1-80 ------MPNEINRGPLPDPWRPVNITVPVTVANDFDQMQSVIKDVMTQLGCPTCHSGRDLRINIATDFV-----VNPA-GQVHQAVGFSFGG----
WP_266323190.1/1-77 ---------MINRGPLPDPWRPVNITVPVSVANDFDQMQSVIKEVMTQLGCPTCHSGRDLRINIATDFV-----VNPA-GQVQQAVGVSFGG----
NZA27150.1/1-80 MK---STTAMNAA-SLRSPHAVTVSVPASVANDLGKMNEVTKSILGRLGCEGCHSGFDIRYLIERDFR-----VNPD-LRIEGFSPHVGF-----
WP_166056965.1/1-79 MG---RKIA-AAA-YMLTPNTVNVSVPAGVANDLKQMNELTKKILGRLGCLGCHSGYDIRFLIERDFR-----VNPQ-LEIEGFNARFGF-----
RSZ60828.1/1-86 MDS-NAVDLRTTLA-AQPATRAVSVSLPSQVYFDLDQFQRVQKELLGQLGCPACTSGFDIRYDIQRRFV-----VDAS-LSVQASGLAQDPLSP--
WP_104140055.1/1-75 MP-------EKDLT-NEFSARTVHVTVPAEVAFNLDRIQTVQKEILGRLGCSACCSGFDIRFDIARRFV-----VDTD-LQVQPAGLLE-------
MBN9380611.1/1-81 M--------ATSDNTHVPASNVVRVTIPASVAYNFDSITKVTQTVLGKLGCLGCHSGRDIRFIIEDNFA-----VDEK-LNVFPNINNVGNELAR-
RLW51783.1/1-81 ME---YPTAIALDKDALRSNVVRVTIPAKAMFDLKKMQKITESVLDQLGCSNCHSGHDIRFDIERQFI-----VDEK-LKIHSQMELMRR-----
WP_079686322.1/1-85 M-----KKLKRNFEPVTSSPPIVRVTVPASVAYNFEQLTSVTQKILAQLGCAPCHSGRFILYEIEDRFV-----VNEN-LEVRPIFDDVRINEKVF
WP_233773391.1/1-77 MKD-AIKDVRLTHG-PTVIDKTIRISVPVKVANNLDQMNKITASVLNQLGCPGCHSGFDLRFDIERQFA-----FDEK-LNPIGH----------
WP_073406981.1/1-72 ---------MATER-KFSKATPIYVSVPSKMS--LEQTHEVTKKILGLVGCPGCYSGFHFQFIDEIEMITAR--IDAKGINVGPSF---------
WP_185801498.1/1-75 ------MPKELTAS-RAIAATPIRVSLPANVAYDFDKFQKVQKDILGRLGCLACCSGWDIRWDIFRDFR-----VDEK-LNVEQGLFR-------
WP_165267142.1/1-79 MK---ISKEDLNSR-PSKKDTAVHVTVPAKVAYNLDEMQKVTSIVLDRLNCPACHSGFDIRFDIEKRFN-----FNED-LELIDRPRFR------
WP_252793625.1/1-72 ---------MAT-R-KFSKATPIYVSVPPKMT--IDQTHAVTKEILGLVGCPGCYSGFHFQFIDETEMIHAR--VDAKGINVGGAQF--------
WP_146742367.1/1-78 ------MGIEMKHALATQKDNIVRVSVPAEILFDFKKFAGIQKDILGRLGCAACTSGHDIRWDITRNFV-----VDIK-GQIHESAPRG------W
WP_096355477.1/1-70 ---------MKSGR-KFSKENAVLVSVPSKLS--LEQTQHITKEILGIVGCPSCYSGFKFQFIDEGEIITARFSENQE-LSIH-----------
MCG8488249.1/1-81 ME---YSTAIALDKDALRSNVVRVTIPAKAMFDLKKMQKITESVLDQLGCGNCHSGHDIRFDIEKQFI-----VDEK-LKVHSQVELMRR-----
MBI4997462.1/1-81 ME---KATSFALQRSEFSANVVRVTIPASAAYDLKQMQKITASILDRLGCSNCHSGHDIRFDLEREFI-----VDAK-LNVHARSELLRG-----
Conservation
---------20303-0002234929 79*537202833632763299559 7*26*5**5385743325 83-----3813-746100000--------

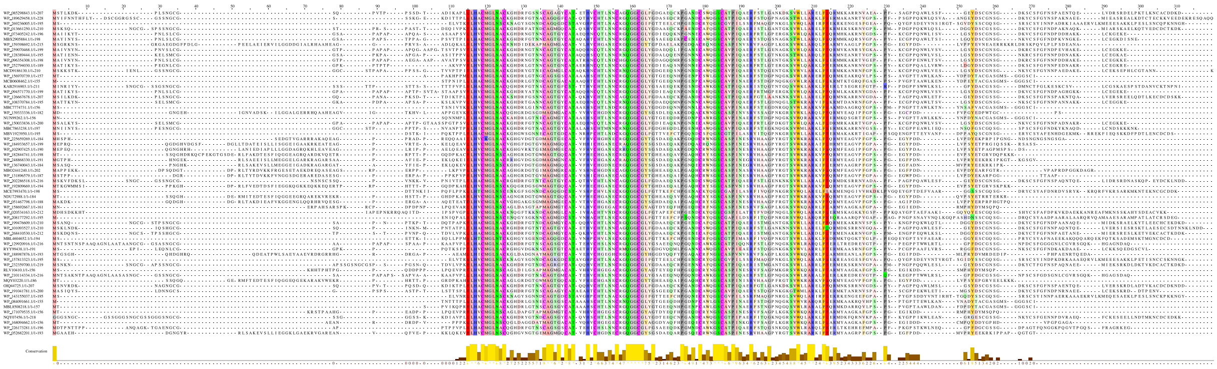

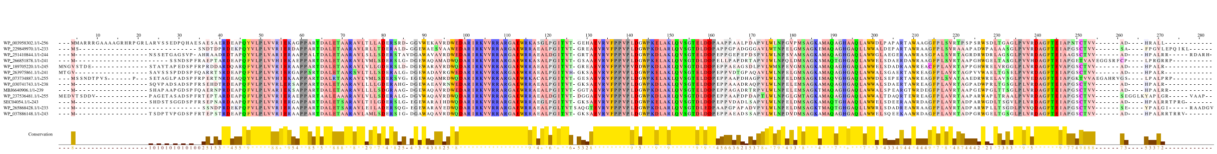

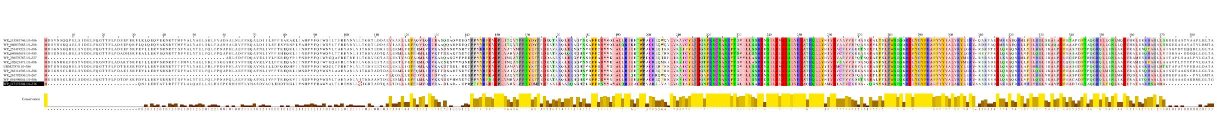

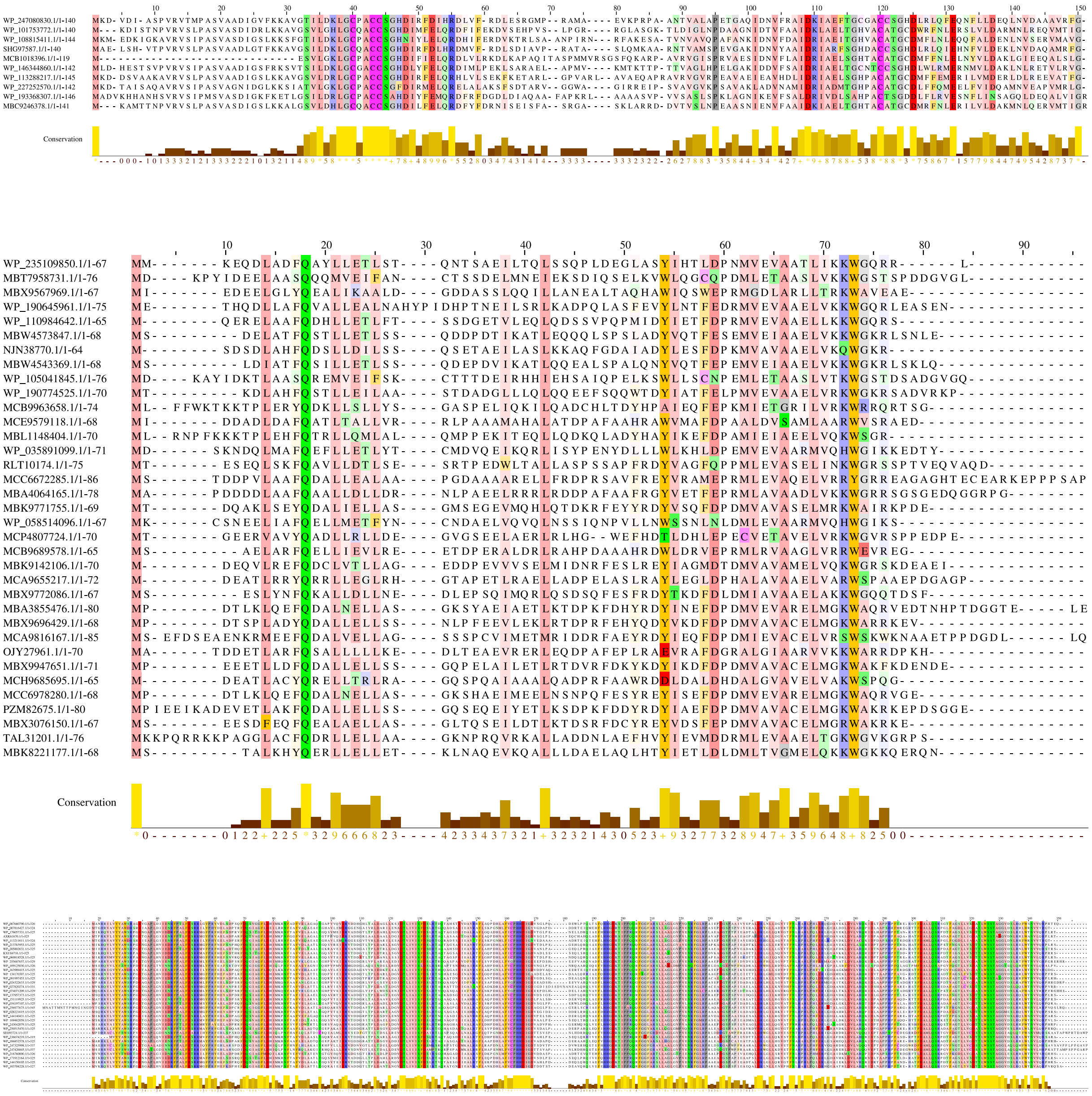

Figure S3. Sequence alignments of new families of proteins. From top to bottom, the alignments shown correspond to the protein families called CxxxxC, adjamnio-1,-2, -3, -7, and adjamnio-I to VI described in the text, respectively. The adjamnio-1, -2, -3, -7, -I and -IV families are probably new precursor families. In contrast, the adjamnio-II, -III, V and VI are most likely not precursors (see text) but contain proteins of unknown functions.

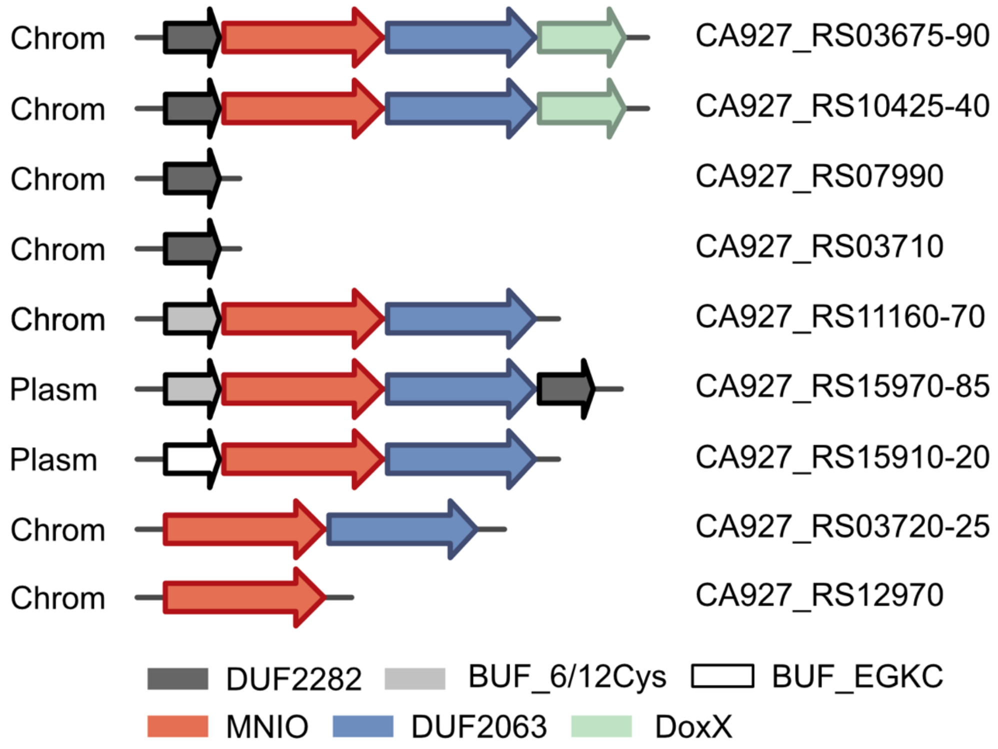

Figure S4. Bufferin-type BGCs in the genome of *Legionella pneumophila subsp fraseri*. The locus tags are provided at the right. Two BGCs are found on a mega plasmid. No genes coding for ECF sigma factors and anti-sigma proteins were found, and no other conserved genes were identified. Note that the bufferin associated with locus tag CA927_RS12970 is encoded by a truncated gene (not shown), suggesting loss of function of this BGC. The BGC of CA927_RS07990 also contains genes coding for a YceI protein (small beta barrel protein described to bind hydrophobic molecules) and a cytochrome B protein. The RiPP precursor of the CA927_RS03710 locus is in translational coupling with a guanylate cyclase domain. 'Chrom' and 'Plasm' indicate the location (on the chromosome or on a plasmid) of the various BGCs.

Files S1-S3. HMM profiles for the new families defined in this work (.hmm files). As we could not upload files with a '.hmm' extension in the submission site, these files will be available upon request.
